# Sleep quality and risk of coronary heart disease - a prospective cohort study from the English longitudinal study of ageing

**DOI:** 10.18632/aging.103866

**Published:** 2020-11-16

**Authors:** Chenxi Song, Rui Zhang, Jiaqiang Liao, Rui Fu, Chunyue Wang, Qianqian Liu, Weihua Song, Hongjian Wang, Kefei Dou

**Affiliations:** 1 Department of Cardiology, Fuwai Hospital, National Center for Cardiovascular Diseases, Chinese Academy of Medical Sciences and Peking Union Medical College, Beijing, China; 2 Department of Environmental and Occupational Health, West China School of Public Health, Sichuan University, Chengdu, China

**Keywords:** sleep quality, sleep duration, the elderly, coronary heart disease

## Abstract

Background: The association between sleep quality and risk of coronary heart disease (CHD) remains unclear in the elderly.

Results: At eight-year follow up, a total of 411 (4.29%) participants developed CHD. Compared with good quality group, the multivariable hazard ratio [HR] (95% confidence interval [CI]) for CHD was 1.393 (1.005, 1.931) for intermediate quality group and 1.913 (1.206, 3.035) for poor quality group. Consistent results were observed in participants with normal sleep duration.

Conclusions: Poor sleep quality may be a novel modifiable risk factor for CHD in the elderly independent of conventional cardiovascular risk factors, even when sleep duration was normal.

Methods: The current study included 9570 CHD-free participants in the English Longitudinal Study of Ageing (ELSA) from wave 4 (2008 to 2009). Incident CHD included new onset angina or myocardial infarction. Sleep quality was measured by a four-item questionnaire. Score ranged from 1 (best) to 4 (poorest). Participants were divided into three groups: good quality (1 ≤ score <2), intermediate quality (2 ≤ score <3) and poor quality (3 ≤ score ≤4). Cox regression model was used to calculate HR for CHD risk according to sleep quality, adjusted for conventional CHD risk factors and sleep duration.

## INTRODUCTION

The number and proportion of people aged 60 years or older has been increasing globally over recent decades [1]. It is estimated that the number of people aged > 60 years will increase from 1 billion in 2019 to 1.4 billion by the end of 2030, and to 2.1 billion by the end of 2050. Poor sleep quality is a common concern in the elderly population aged 75 years or older [2], and affects many aspects of well-being and quality of life, including both physical and mental health conditions [3]. In the research field of sleep quality and coronary heart disease (CHD) risk, the majority of previous studies enrolled middle-aged population, with the mean age ranging from 45 to 58 years [4–8]. Sleep quality was mainly assessed by one or two simple questions, and the assessment methods varied across studies. Inconsistent conclusions were found in previous studies, while poor sleep quality was associated with increased CHD risk in some studies [4, 6, 8], this association was not significant in other studies [5, 7]. In conclusion, the association between sleep quality and CHD risk in the elderly population remains unclear.

The primary objective of the current study is to evaluate the association between sleep quality and CHD risk based on a large cohort study of the elderly population over 8-year follow-up. In addition, since sleep duration is closely related to both sleep quality and CHD risk, we also evaluate whether the association between sleep quality and CHD risk still exists in participants with normal sleep duration.

## RESULTS

### Baseline characteristics

**Figure 1:**
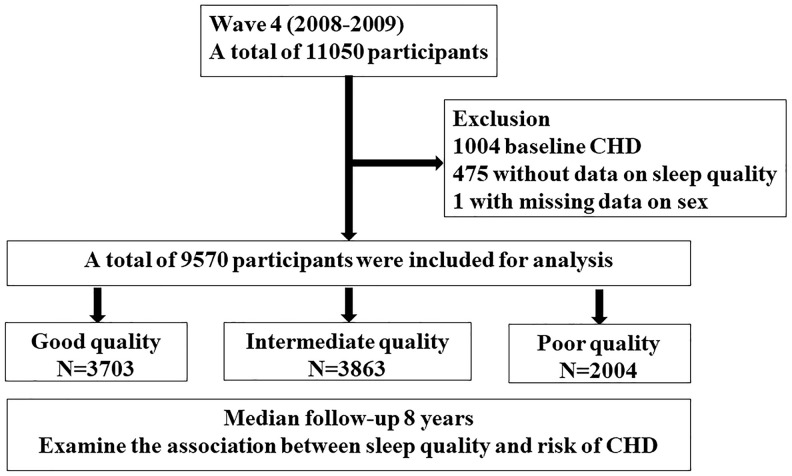
**Study flow chart A total of 11050 participants from English longitudinal study of ageing cohort in wave 4 (2008-2009) were screened.** After exclusion of participants with coronary heart disease at baseline, with missing data on sleep quality and sex, a total of 9570 participants were included for analysis. Participants were divided into three groups according to sleep quality and followed up over 8 years.

Of the 9570 participants included for analysis, the number of participants in good, intermediate and poor sleep quality group was 3703 (38.7%), 3863 (40.4%), and 2004 (20.9%) respectively (Figure 1). Mean age of our study cohort was 64.38 years, and the proportion of female was 57.1%. The proportion of female was highest in poor quality group (70.8%), followed by intermediate (57.6%) and good quality (49.2%) group. The proportion of depression symptom was also highest in poor quality group (10.4%), followed by intermediate group (3.0%) and good quality group (1.0%). Compared with good quality or intermediate quality group, poor quality group were less likely to drink alcohol greater than once a week, and less likely to have physical activities. The proportion of participants with prior hypertension increased progressively as sleep quality decreased (good, intermediate and poor sleep quality: 46.1%, 48.5% and 49.0%, respectively). Similarly, the proportion of participants with prior diabetes increased progressively as sleep quality decreased (good, intermediate and poor sleep quality: 12.5%, 14.1% and 15.0%, respectively).

No significant difference in wealth, marital status, education level, NS-SEC classification, BMI, medical history of high cholesterol, chronic obstructive pulmonary disease and cancer were found across groups (Table 1).

**Table 1:** Baseline characteristics according to sleep quality.

**Variable**	**Good Quality N=3703**	**Intermediate Quality N=3863**	**Poor quality N=2004**	**P value**
**Age (years)**	64.5±10.3	64.9 ±10.0	63.2±9.8	<0.001
**Women (%)**	1822 (49.2)	2224 (57.6)	1418 (70.8)	<0.001
**Wealth quintile (%)**				0.201
**1 (lowest)**	548 (17.1)	627 (18.7)	313 (18.1)	
**2**	646 (20.2)	630 (18.8)	366 (21.1)	
**3**	642 (20.0)	649 (19.3)	3587 (20.7)	
**4**	654 (20.0)	727 (21.7)	341 (19.7)	
**5 (highest)**	715 (22.3)	725 (21.6)	356 (20.5)	
**NS-SEC classification (%)**				0.622
**1**	1028 (31.9)	1018 (30.3)	548 (31.7)	
**2**	794 (24.6)	842 (25.1)	412 (23.8)	
**3**	1402 (43.5)	1495 (44.6)	768 (44.4)	
Education^*^ (%)				0.246
**≥NVQ3/GCE A level**	1518 (41.0)	1579 (40.9)	779 (38.9)	
**Marital status (%)**				0.361
**First and the only marriage**	2077 (56.1)	2122 (54.9)	1087 (54.2)	
**BMI (kg/m^2^)**	27.3±7.1	27.4±7.4	26.9±7.6	0.122
**Depression symptoms (%)**	34 (1.0)	96 (3.0)	126 (10.4)	<0.001
**Current smoking (%)**	514 (15.5)	472 (13.9)	392 (22.1)	<0.001
**At least one alcoholic drink/week (%)**	2133 (66.5)	2157 (63.9)	876 (52.0)	<0.001
**At least one moderate activity/week (%)**	3004 (81.1)	3053 (79.0)	1309 (65.3)	<0.001
**Hypertension (%)**	1708 (46.1)	1873 (48.5)	982 (49.0)	0.049
**Diabetes (%)**	461 (12.5)	545 (14.1)	300 (15.0)	0.017
**High cholesterol (%)**	1206 (32.6)	1248 (32.3)	632(31.5)	0.725
**COPD (%)**	95 (2.6)	175(4.5)	138 (6.9)	<0.001
**Cancer (%)**	168 (4.5)	197 (5.1)	107(5.3)	0.337

NS-SEC: national statistics social-economic classification;1: managerial and professional occupations; 2: Intermediate occupations; 3: routine and manual occupations; NVQ: National Vocational Qualification; GCE: General Certificate of Education; CSE: Center for Continuing Education; BMI: body mass index; COPD: chronic obstructive pulmonary disease.

^*^education: NVQ3/GCE A level is equivalent to senior high school.

### Sleep quality and outcome

A total of 411 (4.3%) incident cases occurred at a median 8-year follow-up. The number (incidence) of incident CHD cases in good, intermediate and poor quality groups was 128 (3.5%), 174 (4.5%) and 109 (5.4%), respectively. After simple adjustment of age and sex (model 1), the risk of CHD increased progressively with decreased sleep quality (Table 2). Compared with good quality group, HR (95% CI) for intermediate quality group was 1.331 (1.059, 1.674), for poor quality group was 2.001 (1.542, 2.596). Further adjustment of social-economic status, life style and conventional CHD risk factors did not significantly alter the association. Compared with good quality group, HR (95% CI) for intermediate quality group was 1.393 (1.005, 1.931), for poor quality group was 1.913 (1.206, 3.035). When the sleep quality score was modeled as continuous variable, one score increase was significantly associated with increased risk of CHD in all three models (Table 2).

**Table 2:** Association between baseline sleep quality with risk of CHD.

	**Categorical**			**Continuous**
	**Good Quality N=3703**	**Intermediate Quality N=3863**	**Poor quality N=2004**	**Per one score increase**
No. of Cases /Person-Years	128/22596	174/24090	109/11824	411/58510
Model 1	1 (Reference)	1.331 (1.059, 1.674)	2.001 (1.542, 2.596)	1.444 (1.279, 1.630)
P value		0.014	<0.001	<0.001
Model 2	1 (Reference)	1.296 (0.998, 1.684)	1.889 (1.399, 2.553)	1.431 (1.243, 1.646)
P value		0.052	<0.001	<0.001
Model 3	1 (Reference)	1.393 (1.005, 1.931)	1.913 (1.206, 3.035)	1.502 (1.209, 1.865)
P value		0.047	0.006	<0.001

Model 1: adjusted for age, sex

Model 2: model 1+education, social economic classification, marital status, income

Model 3: model 2+ current smoking, alcohol consumption, BMI, physical activity, diabetes, high blood cholesterol, hypertension, depression, sleep duration

The association between baseline sleep quality with secondary outcome are shown in Supplementary Tables 1 and 2, respectively. In all three models, the risk of angina increased progressively as sleep quality decreased. Regarding MI, intermediate quality group had a trend towards higher risk of MI compared with good quality group, but the association was not significant in all three models. Poor quality group had a significant higher risk of MI after adjustment of age and sex (model 1) and socio-economic factors (model 2), and a trend towards higher risk of MI after further adjustment of conventional CHD risk factors plus sleep duration (model 3, Supplementary Table 2).

The distribution of sleep quality score change between wave 4 and wave 6 are shown in Supplementary Figure 1. Approximately 90% of participants had a change less than or equal to 1 point, which indicated that a majority of participants in our study did not show a significant change in sleep quality during the first 4 years. Compared with maintaining good sleep quality between wave 4 and wave 6, maintaining intermediated sleep quality, and quality worsened was associated with trend towards higher CHD risk, and maintaining poor quality was associated with significant higher CHD risk in fully adjusted model (HR: 1.876, 95% CI: 1.055, 3.334, Supplementary Table 3). No obvious trend or significant association was found for sleep quality improved (HR: 0.992, 95% CI: 0.606, 1.624).

We also evaluated the association between the frequency of each sleep problem and risk of CHD (Supplementary Table 4). We have studied three sleep problems: difficulty falling asleep and wake up several times at night and wake up feeling tired and worn out. Regarding difficulty falling asleep, compared with patients who did not report the problem during the last month, those reported “three or more times a week” had higher risk of CHD (HR: 1.843, 95% CI: 1.166, 2.913, p=0.009). Similarly, participants who reported “wake up several times at night” three or more times a week had higher risk of CHD (HR: 1.710, 95% CI: 1.153, 2.538, p=0.008). Regarding “wake up feeling tired and worn out”, a frequency of “once or twice a week” (HR: 1.570, 95% CI: 1.022, 2.412, p=0.039) and “three or more times a week” (HR: 1.921, 95% CI: 1.280, 2.881, p=0.002) was associated with increased CHD risk.

In order to assess whether sleep quality improved the predictive performance of a tradition risk score, we compared the C-statistics between the traditional Framingham 10-year CVD risk score, and traditional+sleep risk score (new risk score). Framingham 10-year CVD risk score was developed based on age, sex, diabetes status, smoking status, treated or untreated systolic blood pressure, and blood lipids to predict 10-year CVD risk in the Framingham study [9]. C statistics for the new and traditional risk score was 0.66 and 0.65, respectively (C-statistics difference: 0.01, 95% CI: 0.0004-0.03, p=0.04, Supplementary Figure 2). Integrated Discrimination Improvement (IDI) was 0.003 (95% CI: 0.002, 0.005, P<0.001) and Net Reclassification Improvement (NRI) was 0.203 (95% CI: 0.095,0.311, P<0.001).

### Subgroup and sensitivity analysis

Subgroup analysis showed sleep quality-CHD risk association did not differ significantly according to age, sex or depression symptoms (P interaction: 0.322 for age; 0.783 for sex, 0.657 for depression symptom respectively Table 3). In each age subgroup, poor sleep quality had a trend towards higher risk of CHD compared with good sleep quality. A similar trend was found in male, female, depression or no depression subgroup.

**Table 3:** Association between baseline sleep quality and risk of coronary according to age, sex and depression.

	**Good quality**	**Intermediate quality**	**Poor quality**	**P _interaction_**
**Age**				0.322
<60 (N=3462)	1 (Reference)	1.007 (0.497, 2.043)	1.433 (0.598, 3.433)	
[60-70) (N=3278)	1 (Reference)	2.000 (1.110, 3.603)	2.192 (1.000, 4.804)	
[70-80) (N= 2027)	1 (Reference)	1.158 (0.685, 1.957)	1.449 (0.655, 3.202)	
≥80 (N=803)	1 (Reference)	1.905 (0.619, 5.866)	5.837 (1.740, 19.576)	
**Sex**				0.783
Male (N=4106)	1 (Reference)	1.315 (0.878, 1.970)	1.665 (0.873, 3.176)	
Female (N=5464)	1 (Reference)	1.562 (0.897, 2.719)	2.246 (1.177, 4.285)	
**Depression symptom**				0.657
Yes (N=256)	1 (Reference)	1.466 (0.150, 14.326)	1.010 (0.103, 9.869)	
No (N=7570)	1 (Reference)	1.385 (0.995, 1.927)	2.004 (1.255, 3.199)	

adjusted for age, sex, education, social economic classification, marital status, income, current smoking, alcohol consumption, BMI, physical activity, diabetes, high blood cholesterol, hypertension, depression, sleep duration

To reduce the effect of missing data, we used multiple imputation and calculated the HR (95% CI) for each outcome in the imputed dataset (Supplementary Table 5). Results in the imputed dataset were general consistent with the main analysis. Compared with good sleep quality, intermediate quality (HR: 1.385, 95% CI:1.028, 1.864, p < 0.001) and poor quality (HR: 1.945, 95% CI: 1.285, 2.943, p < 0.001) was associated with increased CHD risk, which was mainly driven by increased angina risk but not MI.

Since sleep duration is closely related to sleep quality, we also examined the effect of sleep quality on CHD risk in participants with normal sleep duration. Sensitivity analysis was performed by excluding participants with too short (<6 hours) or too long sleep (>9 hours). Poor sleep quality was associated with higher CHD risk than good sleep quality in fully adjusted model (HR: 2.058, 95% CI: 1.230, 3.444, p=0.006). Per one score increase was associated with 54.2% increased risk in CHD (Table 4).

**Table 4:** Association between baseline sleep quality with risk of CHD in participants with normal sleep duration (6–9 hours).

	**Categorical**			**Continuous**
	**Good Quality N=3466**	**Intermediate Quality N=3397**	**Poor quality N=1168**	**Per one score increase**
No. of Cases /Person-Years	120/21292	150/21370	57/6970	327/49632
Model 1	1 (reference)	1.313 (1.032, 1.671)	1.813 (1.316, 2.498)	1.416 (1.216, 1.648)
P value		0.027	<0.001	<0.001
Model 2	1 (reference)	1.239 (0.940, 1.634)	1.654 (1.139, 2.403)	1.364 (1.143, 1.628)
P value		0.129	0.008	<0.001
Model 3	1 (reference)	1.353 (0.958, 1.911)	2.058 (1.230, 3.444)	1.542 (1.212, 1.962)
P value		0.086	0.006	<0.001

Model 1: adjusted for age, sex

Model 2: model 1+education, social economic classification, marital status, income

Model 3: model 2+ current smoking, alcohol consumption, BMI, physical activity, diabetes, high blood cholesterol, hypertension, depression, sleep duration

## DISCUSSION

In this prospective large-scale elderly cohort of participants, poor sleep quality was associated with a 91.3% increased risk of developing CHD than good sleep quality, after adjustment of demographic, socio-economic, conventional CHD risk factors and sleep duration. There was also a positive relationship between frequency of each individual sleep problem and CHD risk. The sleep quality-CHD risk association was consistently observed in various subgroups according to age, sex or the presence of depression symptoms. What’s more important, for participants with normal sleep duration, poor sleep quality was associated with increased risk of CHD than good sleep quality.

### Comparison with previous studies

The differences of between our study and previous studies mainly fall into the following two aspects: First, we investigated the sleep quality and CHD risk’s association in the elderly population. There have been a few studies examined the association between sleep quality and CHD risk [4–8]. However, the mean age in these studies ranged from 45 to 58 years, while the mean age of our study was 64.4 years old. The evidence regarding sleep quality and CHD risk’s association in the elderly remains limited. To address this issue, our study used data from the English Longitudinal Study of Ageing, which enrolled participants aged 50 years or more, with a mean age of over 64 years, and examined the sleep quality-CHD risk association. Second, to our knowledge, all previous studies assessed sleep quality only once at baseline, which may not be precise since sleep quality can change over time. By comparison with previous reports, our study not only analyzed the association between baseline sleep quality and CHD risk, but also used data from two sleep assessments at wave 4 and wave 6 to examine the effect of sleep quality change pattern on CHD risk, which provided a more accurate and comprehensive assessment of sleep quality status. Our findings suggested that compared with maintaining good sleep quality, all other quality change patterns (quality improved or worsened, or maintaining intermediate quality) were associated with a trend towards higher risk, and maintaining poor sleep quality was associated with a significant higher CHD risk, highlighting the clinical significance of maintaining good sleep quality.

Methods of sleep quality assessment, and the corresponding definition of “good quality” varied across studies, ranging from one single question to sleep questionnaire including 10 items, which makes direct comparison difficult. However, the assessment methods in our study are in general consistent with previous studies, and covered the three main area, which includes difficulty falling asleep, difficulty maintaining sleep and waking up feeling worn out.

### Explanations for the association between sleep quality and CHD risk

The biological mechanisms underlying poor sleep quality and CHD risk remain unclear, but previous studies suggested several possible explanations. First, sleep disturbance was associated with conventional CHD risk factors including diabetes [10], hypertension [11] and obesity [12]. Second, previous studies suggested that chronic sleep disturbance was associated with sustained inflammation activation, which plays a key role in the pathophysiology of CHD [13]. A large-scale meta-analysis including over 50000 participants revealed that sleep disturbance was associated with elevated inflammatory biomarkers including CRP and IL-6 [14]. Third, lipid metabolism may be an important pathogenic pathway linking poor sleep quality and CHD risk. Lipid metabolism disorder is an important cause of CHD. Previous studies demonstrated that lipid and energy metabolism in human were regulated by circadian system [15]. One nested case-control study found 9 lipid metabolites were higher in woman with poor sleep quality after adjusting age, race, BMI and smoking, and a metabolite score derived from the above 9 metabolites were positively associated with CHD risk after adjusting conventional CHD risk factors [16]. Finally, the hypothalamic-pituitary-adrenocortical (HPA) axis activation and subsequent altered diurnal cortisol levels may also contribute the association between sleep quality and CHD risk [17].

Of note, we observed a significant association between poor sleep quality and angina pectoris, but not myocardial infarction in the fully adjusted model. Possible explanations include: First, the pathophysiology underlying angina pectoris and myocardial infarction were different. Angina pectoris was mainly caused by episodes of reversible myocardial demand/supply mismatch, when the demand is greater than supply and is often induced by exercise, emotion or stress [18]. Myocardial infarction (MI) is defined in pathology as myocardial cell death due to prolonged myocardial ischemia [19], mainly caused by the rupture of atherosclerosis plaque and subsequent blockage of coronary artery. The induced condition for angina in daily life may be more easily achieved compared with myocardial infarction. Second, some participants who developed AMI may die before the outcome data was collected. While we assessed non-fatal MI, we were unable to assess the association between sleep quality and fatal MI because we currently don’t have access to mortality data, including survival status and detailed causes of death during follow-up. Finally, the limited number of cases and relatively short follow-up period may explain the non-significant association between sleep quality and AMI.

### Clinical significance

Poor sleep quality is a common problem in the elderly population. Our study found that poor sleep quality was associated with increased CHD risk, even when sleep duration was normal, and suggested that sleep disorders as novel modifiable risk factors for incidence CHD. Sleep quality may be taken into consideration in future CHD risk assessment, and health education is needed to increase public awareness of poor sleep quality and its potential adverse health effects, in order to promote timely consultation with medical staffs. However, it is worthy to note that our study was an observational study, and more future studies are needed to firstly validate the causal relationship between poor sleep quality and CHD risk, and whether current interventions targeting sleep problems, including psychological treatment and medical devices, are effective in reducing CHD risk.

### Strengths and limitations

The current study used data from a well-established elderly cohort with 8 years of follow-up. Our findings provide additional evidence regarding sleep quality-CHD risk association in the elderly population. We further validate our findings by mitigating the bias of abnormal sleep duration. We adjusted sleep duration in the multivariate regression model. In addition, we performed sensitivity analysis by excluding those with abnormal sleep duration, and examined sleep quality-CHD risk association in participants with 6–9 hours of sleep duration.

There are several limitations in the study. Firstly, sleep quality was self-reported and may subject to reporting and selecting bias. We were unable to take into account objective measures of sleep, use of certain sleep medication, or clinical diagnoses such as sleep apnea or insomnia syndrome because these data were not collected in the cohort. However, we found self-reported poor sleep quality, though not an objective sleep measurement, was indeed associated with increased CHD risk after adjustment of confoundings, suggesting an important role of subjective feeling of “good sleep” in cardiovascular health. Second, angina was one component of the primary outcome, which was relatively subjective and may introduce reporting bias. However, angina pectoris was diagnosed by physicians and previous studies supported that angina symptoms was a predictor for future adverse cardiovascular events [20] and impaired quality of life [21]. Therefore, it is of clinical significance to find novel modifiable risk factors for angina pectoris in order to improve participants’ outcome and quality of life. Third, we currently do not have access to data regarding survival status or causes of death during follow-up, so we were unable to investigate the association between sleep quality and fatal myocardial infarction in the current study. Fourth, time to disease onset was calculated based on the first wave of newly onset disease, but not the exact date. Since follow-up was carried out every two years, the time period free of CHD may not be accurate. Fifth, although we adjusted for multiple confounders, there might be unmeasured and residual confounders. Sixth, we included baseline covariates in the multivariable model, but did not consider the serial change of covariates. Finally, our results are not generalizable to younger age-groups and other ethnicities. However, the main objective of the current study is to investigate the sleep quality-CHD risk association in the elderly.

## CONCLUSIONS

Poor sleep quality is associated with increased risk of CHD, even when sleep duration is normal and after multivariate adjustment in the older population. Our findings suggested that poor sleep quality as novel modifiable risk factors for CHD due to biological plausibility, and future studies are required to explore whether sleep intervention strategy is effective on CHD risk reduction.

## METHODS

### Participants

We used data from the English longitudinal study of ageing (ELSA), a well-established prospective cohort of participants aged >50 years old living in the UK. Detailed description of the cohort was published previously [22]. In brief, participants in this cohort were followed up every 2 years since 2002, and data were collected regarding family and job, social and economic issues, physical and mental health, behavior and cognition, etc. On wave 2, 4, 6, 8, a nurse visit was carried out to collect biological samples and assess anthropometric measurements, such as body mass index (BMI). The current study used data from wave 4 (2008) to wave 8 (2016). Wave 4 was selected as baseline since this is the first wave when data on sleep characteristics were collected. Study flow chart is shown in Figure 1. We excluded participants who reported angina or heart attack at wave 4 (N=1004), those with missing data on sleep quality (N=475) and sex (N=1), and the remaining 9570 participants were included for analysis. The authors assert that all aspects of this work follows the World Medical Association's Declaration of Helsinki. All participants were provided informed consent and the project received ethical approval from the National Research Ethics Service.

### Exposures

Sleep quality is the exposure of the current study. Sleep quality was assessed by the following 4 questions adapted from Jenkins Sleep Scale [23]: 1) How often respondent has difficulty falling asleep; 2) Frequency wake up several times at night; 3) Frequency wake up feeling tired and worn out; 4) Rating sleep quality overall. Jenkins sleep scale was validated in previous clinical samples and large-scale cohort study [24]. The Cronbach’s alpha was 0.73 at wave 4 of our study population. Regarding the first three questions, respondents answered the frequency of each sleep problem, including 1) Not during the last month (score=1); 2) Less than once a week (score=2); 3) Once or twice a week (score=3); 4) Three or more times a week (score=4). Regarding the fourth question, participants will rate overall sleep quality as 1) very good (score=1); 2) good (score=2); 3) fairly bad (score=3); 4) very bad (score=4). A sleep quality score was calculated by averaging the score of the above four questions [25]. The sleep quality score range from 1 (best sleep quality) to 4 (worst sleep quality).

Participants were divided into three groups according to the sleep quality score: 1) Good quality (1<score<2); 2) Intermediate quality (2≤score<3); 3)Poor quality (3≤score≤4) [25]. Sleep duration was self-reported number of hours on average week night.

To explore the association between sleep quality change and incident CHD risk, participants were divided into five groups according to the sleep quality in wave 4 and wave 6: 1) Maintaining good sleep quality group (good sleep quality in both wave 4 and wave 6); 2) Quality improved group (good sleep quality in wave 6 and intermediate/poor quality in wave 4, or intermediate quality in wave 6 and poor quality in wave 4); 3) Quality worsened group (poor quality in wave 6 and good/intermediate quality in wave 4 or intermediate quality in wave 6 and good quality in wave 4); 4) Maintaining intermediate group (intermediate quality in both wave 4 and wave 6); 5) Maintaining poor quality group (poor quality in both wave 4 and wave 6). Sleep quality score change as a continuous variable was also calculated by subtracting wave 4 sleep quality score from wave 6 sleep quality score.

### Outcome

The primary outcome of the current study is coronary heart diseases, defined as a composite endpoint of self-reported physician diagnosed angina or MI in computer assisted personal interviewing. Participants were shown a list of illnesses and asked to report any doctor-diagnosed illness they had received. Incident angina (or MI) was defined as a positive report of physician-diagnosed angina (or heart attack) from wave 5 to wave 8. Participants reported angina or MI at baseline (wave 4) were excluded. Survival time, which was the time from baseline to angina or MI onset, was calculated based on the first wave when disease was reported. For instance, if a participant reported angina on wave 6 for the first time, then survival time was the time interval between wave 4 and wave 6 (equal to 4 years because time interval between each consecutive two waves was 2 years). If a participant was lost to follow-up before wave 8 and did not report angina or MI, then survival time was calculated as the time from baseline to the wave of last follow-up. Secondary outcome in the current study was defined as individual component of the primary outcome, namely 1) incident angina; 2) incident MI.

### Covariates

Covariates are selected based on their possible association with CHD [26], including age, sex, wealth, social class, education, marital status, depression, current smoking, alcohol intake, BMI, physical activity, medical history of diabetes, hypertension, high cholesterol and sleep duration. Wealth was defined as total non-pension wealth, which combines total financial, physical, and housing wealth but excluded pension wealth, and categorized in quintiles. Social class was categorized by using National Statistics-Socio Economic Classification (NS-SEC), and included: 1) managerial and professional occupations; 2) Intermediate occupations; 3) routine and manual occupations. Education was categorized into six groups according to the highest education qualification at wave 4: 1) National Vocational Qualification (NVQ) 4/5/ degree level or equivalent; 2) higher ed below degree; 3) NVQ3/General Certificate of Education (GCE) A level equivalent; 4) NVQ2/GCE O level or equivalent; 5) NVQ1/ Center for Continuing Education (CSE) or other grade equivalent; 6) foreign/no qualification/ others. Marital status was categorized into seven groups: 1) single; 2) married first and only marriage; 3) remarried; 4) legally separated; 5) divorced; 6) widowed; 7) civil partner/others. Depression symptoms were assessed by Centre for Epidemiological Studies Depression scale (CES-D), and a score≥4 indicated depression symptoms [27]. Physical activity was categorized into 2 groups according to whether moderate or vigorous activity once or more per week. Alcohol intake was categorized into 2 groups according to whether drink alcohol once or more per week. Hypertension was defined as systolic blood pressure greater than 140mmHg and/or diastolic blood pressure greater than 90mmHg, or self-reported hypertension medical history or using anti-hypertensive drugs. Diabetes was defined as HbA1c ≥ 6.5% or fasting blood glucose >7mmol/L or self-reported diabetes medical history, or using insulin/anti-diabetic medication. Sleep duration was modeled as a continuous variable in the cox regression model.

### Statistical analysis

Continuous variables are presented as mean±SD and compared across groups by using ANOVA test. Categorical variables are presented as count (frequency) and compared across groups by using chi-square test. Cox regression model was used to calculate hazard ratios (HRs) and 95% confidence interval (CI) for incident CHD in relation to sleep quality. We used a total of three cox regression models. We firstly adjusted age and sex (model 1). To examine whether the association was independent of socio-economic factors, we further adjusted education, social economic classification, marital status, income (model 2). Because sleep duration was closely related to sleep quality and CHD risk, the final fully adjusted model (model 3) included all covariates in model 2 plus sleep duration and conventional CHD risk factors (current smoking, alcohol consumption, BMI, physical activity, medical history of diabetes, hypertension). To examine whether sleep quality improved the predictive performance of a tradition risk score, we compared the C-statistics between the traditional Framingham CVD risk score, and the traditional+sleep risk score. Differences in C-statistics of these two models, IDI and NRI was calculated to quantify the improvement in model performance.

Subgroup analysis were performed to examine whether sleep quality-CHD risk association differs according to age (<60, (60–70), (70–80), ≥80) sex (male or female) or depression symptoms (depression or not). Fully adjusted model (model 3) as described above was used for subgroup analysis. Number of missing covariates are shown in Supplementary Table 6. Because most variables had less than 4% missing covariates, we used complete case analysis method as the main analysis. We also used multiple imputation method to handle missing data. We assumed data was missing at random [28] and used the chained equations approach with proc MI procedure in SAS. The number of imputation was 25, and the number of burn-in iterations was 5. Continuous variables were imputed by using regression method, and categorical variables were imputed by using logistic methods. We then performed fully adjusted model (model 3) in each of the 25 imputed datasets. The final estimates were calculated according to the 25-fold imputation procedure. We performed the second sensitivity analysis by excluding participants with sleep duration <6 hours or >9 hours, to examine whether poor sleep quality was associated with increased CHD risk in participants with normal sleep duration. The definition of normal sleep duration (6–9 hours) was chosen in accordance with previous reports [29], and we would like to enroll participants as many as possible and increased the extrapolation of the current study. The fully adjusted regression model (model 3) was used to examine the association with sleep quality and primary outcome in participants with normal sleep duration. All statistical analysis was performed using SAS 9.4 (SAS Institute, Cary, NC, USA).

## Supplementary Material

Supplementary Figures

Supplementary Tables
