# Supplementary material for: Sleep quality and risk of coronary heart disease - a prospective cohort study from the English longitudinal study of ageing
Source: Aging (Albany NY). 2020 Nov 16;12(24):25005–19. doi: 10.18632/aging.103866 (PMC7803507; doi:10.18632/aging.103866)
Supplement: Supplementary Figures [file aging-12-103866-s001.pdf]

SUPPLEMENTARY FIGURES

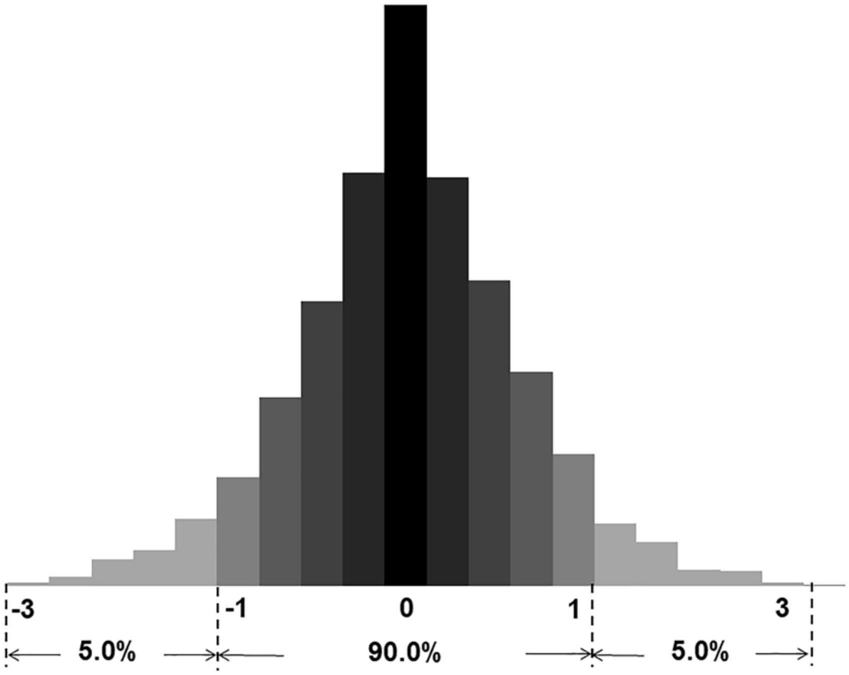

Supplementary Figure 1. Distribution of sleep quality score change between wave 4 and wave 6. Sleep quality score change was calculated by subtracting wave 4 sleep quality score from wave 6 sleep quality score. Approximately 90.0% participants had score change less than or equal to 1 score.

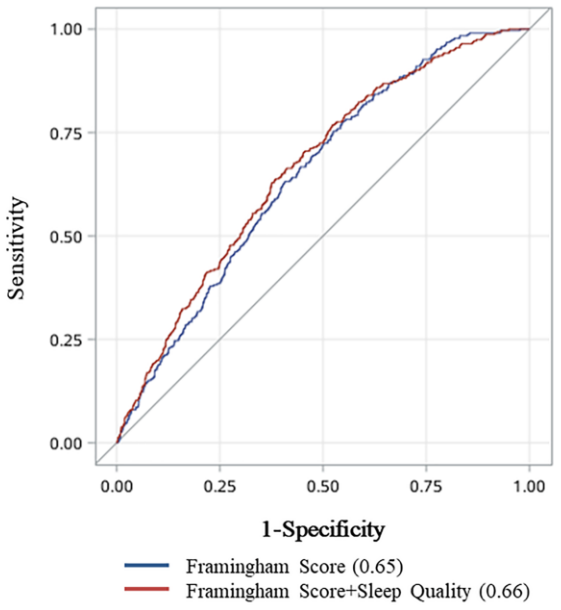

Supplementary Figure 2. Added predictive ability of sleep quality. Receiver operating characteristic curve (ROC) of the Framingham 10-year CVD risk score (blue) and Framingham 10-year CVD risk score plus sleep quality (red) are shown in this figure. Area under ROC curve (AUC) for Framingham score and Framingham score +sleep quality was 0.65 and 0.66, respectively (P=0.04).
