# Supplementary material for: Sleep quality and risk of coronary heart disease - a prospective cohort study from the English longitudinal study of ageing
Source: Aging (Albany NY). 2020 Nov 16;12(24):25005–19. doi: 10.18632/aging.103866 (PMC7803507; doi:10.18632/aging.103866)
Supplement: Supplementary Tables [file aging-12-103866-s002.pdf]

## SUPPLEMENTARY TABLES

**Supplementary Table 1. Association between baseline sleep quality with risk of angina.**

| | Categorical | | | Continuous |
|---|---|---|---|---|
| | **Good Quality N=3703** | **Intermediate Quality N=3863** | **Poor quality N=2004** | **Per one score increase** |
| No. of Cases /Person-Years | 66/22596 | 108/24090 | 70/11824 | 244/58510 |
| Model 1 | 1 (reference) | 1.588 (1.168, 2.159) | 2.428 (1.724, 3.420) | 1.611 (1.378, 1.884) |
| P value | | 0.003 | <0.001 | <0.001 |
| Model 2 | | 1.456 (1.026, 2.066) | 2.162 (1.454, 3.216) | 1.558 (1.297, 1.871) |
| P value | 1 (reference) | 0.035 | <0.001 | <0.001 |
| Model 3 | | 1.585 (1.025, 2.452) | 2.677 (1.497, 4.786) | 1.745 (1.319, 2.310) |
| P value | 1 (reference) | 0.039 | <0.001 | <0.001 |

Model 1: adjusted for age, sex
Model 2: model 1+education, social economic classification, marital status, income
Model 3: model 2+ current smoking, alcohol consumption, BMI, physical activity, diabetes, high blood cholesterol, hypertension, depression, sleep duration

**Supplementary Table 2. Association between baseline sleep quality with risk of myocardial infarction.**

| | Categorical | | | Continuous |
|---|---|---|---|---|
| | **Good Quality N=3703** | **Intermediate Quality N=3863** | **Poor quality N=2004** | **Per one score increase** |
| No. of Cases /Person-Years | 76/22596 | 83/24090 | 54/11824 | 213/58510 |
| Model 1 | 1 (reference) | 1.089 (0.797, 1.487) | 1.742 (1.221, 2.485) | 1.313 (1.107, 1.558) |
| P value | | 0.594 | 0.002 | 0.002 |
| Model 2 | | 1.173 (0.819, 1.681) | 1.726 (1.139, 2.615) | 1.318 (1.083, 1.605) |
| P value | 1 (reference) | 0.384 | 0.010 | 0.006 |
| Model 3 | | 1.279 (0.818, 2.000) | 1.493 (0.764, 2.919) | 1.325 (0.976, 1.797) |
| P value | 1 (reference) | 0.280 | 0.241 | 0.071 |

Model 1: adjusted for age, sex
Model 2: model 1+education, social economic classification, marital status, income
Model 3: model 2+ current smoking, alcohol consumption, BMI, physical activity, diabetes, high blood cholesterol, hypertension, depression, sleep duration

**Supplementary Table 3. Association between sleep quality change and risk of incident CHD.**

| | Cases /Person-Year | Model 1 | Model 2 | Model 3 |
|---|---|---|---|---|
| | | HR (95% CI) | | |
| Maintaining good quality | 73/13244 | 1 (reference) | 1 (reference) | 1 (reference) |
| Quality improved | 63/10262 | 1.202 (0.857, 1.685) | 1.008 (0.682, 1.490) | 0.992 (0.606, 1.624) |
| Maintaining intermediate quality | 122/16942 | 1.298 (0.969, 1.737) | 1.176 (0.848, 1.631) | 1.288 (0.856, 1.939) |
| Quality worsened | 83/11588 | 1.340 (0.978, 1.836) | 1.189 (0.833, 1.698) | 1.051 (0.675, 1.635) |
| Maintaining Poor quality | 70/6474 | 2.430 (1.742, 3.390) | 2.235 (1.530, 3.265) | 1.876 (1.055, 3.334) |

Model 1: adjusted for age, sex

Model 2: model 1+education, social economic classification, marital status, income

Model 3: model 2+ current smoking, alcohol consumption, BMI, physical activity, diabetes, high blood cholesterol, hypertension, depression, sleep duration

**Supplementary Table 4. Contribution of each individual sleep problem at baseline to CHD risk.**

| | Cases/Person-Year | HR (95% CI) | P value |
|---|---|---|---|
| **Difficulty falling asleep** | | | |
| Not during the last month | 237/35330 | 1 (reference) | |
| Less than once a week | 43/7906 | 1.020 (0.642, 1.620) | 0.935 |
| Once or twice a week | 47/6606 | 1.407 (0.886, 2.233) | 0.148 |
| Three or more times a week | 84/8668 | 1.843 (1.166 2.913) | 0.009 |
| **Wake up several times at night** | | | |
| Not during the last month | 76/14020 | 1 (reference) | |
| Less than once a week | 39/6702 | 1.363 (0.789, 2.353) | 0.267 |
| Once or twice a week | 63/10444 | 1.180 (0.718, 1.941) | 0.514 |
| Three or more times a week | 233/27344 | 1.710 (1.153, 2.538) | 0.008 |
| **Wake up feeling tired & worn out** | | | |
| Not during the last month | 172/28810 | 1 (reference) | |
| Less than once a week | 62/9724 | 1.171 (0.769, 1.784) | 0.462 |
| Once or twice a week | 68/8962 | 1.570 (1.022, 2.412) | 0.039 |
| Three or more times a week | 109/11014 | 1.921 (1.280, 2.881) | 0.002 |

Adjusted for age, sex, education, social economic classification, marital status, income, current smoking, alcohol consumption, BMI, physical activity, diabetes, high blood cholesterol, hypertension, depression, sleep duration

**Supplementary Table 5. Association between baseline sleep quality with outcome in imputed dataset.**

| | Categorical | | | Continous |
|---|---|---|---|---|
| | Good Quality | Intermediate Quality | Poor quality | Per one score increase |
| **CHD** | 1 (reference) | 1.385 (1.028, 1.864) | 1.945 (1.285, 2.943) | 1.467 (1.207, 1.783) |
| **P value** | | < 0.001 | < 0.001 | < 0.001 |
| **Angina** | 1 (reference) | 1.529 (1.030, 2.270) | 2.387 (1.399, 4.071) | 1.675 (1.300, 2.159) |
| **P value** | | 0.035 | < 0.001 | < 0.001 |
| **MI** | 1 (reference) | 1.268 (0.840, 1.916) | 1.737 (0.970, 3.109) | 1.310 (0.996, 1.723) |
| **P value** | | 0.259 | 0.063 | 0.054 |

CHD: coronary heart disease; MI: myocardial infarction

Adjusted for age, sex, education, social economic classification, marital status, income, current smoking, alcohol consumption, BMI, physical activity, diabetes, high blood cholesterol, hypertension, depression, sleep duration

**Supplementary Table 6. Missing data proportion of each covariate.**

| Covariate | N (%) |
|---|---|
| Age | 0 (0) |
| sex | 0 (0) |
| Education level | 298 (3.1) |
| NS-SEC | 356 (3.7) |
| Marital status | 1 (0.01) |
| Total wealth group | 1273 (13.3) |
| Drink alcohol frequency | 207 (2.2) |
| Current smoking | 66 (0.8) |
| Physical activity | 13 (0.1) |
| BMI | 1448 (15.1) |
| Depression | 83 (1.2) |
| Diabetes | 4 (0.04) |
| Hypertension | 4 (0.04) |
| High cholesterol | 14 (0.2) |
| Sleep duration | 24 (0.3) |

NS-SEC: national statistics social-economic classification; BMI: body mass index
